# Salvianolic Acid B Alleviates Liver Injury by Regulating Lactate-Mediated Histone Lactylation in Macrophages

**DOI:** 10.3390/molecules29010236

**Published:** 2024-01-01

**Authors:** Shian Hu, Zehua Yang, Ling Li, Qinwen Yan, Yutong Hu, Feng Zhou, Yang Tan, Gang Pei

**Affiliations:** 1 College of Pharmacy, Hunan University of Chinese Medicine, Changsha 410000, China; 201704080130@stu.hnucm.edu.cn (S.H.); 002181@hnucm.edu.cn (L.L.); 20223732@stu.hnucm.edu.cn (Q.Y.); 20223733@stu.hnucm.edu.cn (Y.H.); 004871@hnucm.edu.cn (F.Z.); 2 Key Laboratory of Modern Research of TCM, Education Department of Hunan Province, Changsha 410000, China; 3 Hunan Drug Inspection Center, Changsha 410000, China; hnsypshcyzx@163.com

**Keywords:** macrophages, salvianolic acid B, LDHA, histone lactylation, H3K18la, liver injury

## Abstract

Salvianolic acid B (Sal B) is the primary water-soluble bioactive constituent derived from the roots of *Salvia miltiorrhiza* Bunge. This research was designed to reveal the potential mechanism of Sal B anti-liver injury from the perspective of macrophages. In our lipopolysaccharide-induced M1 macrophage model, Sal B showed a clear dose-dependent gradient of inhibition of the macrophage trend of the M1 type. Moreover, Sal B downregulated the expression of lactate dehydrogenase A (LDHA), while the overexpression of LDHA impaired Sal B’s effect of inhibiting the trend of macrophage M1 polarization. Additionally, this study revealed that Sal B exhibited inhibitory effects on the lactylation process of histone H3 lysine 18 (H3K18la). In a ChIP-qPCR analysis, Sal B was observed to drive a reduction in H3K18la levels in the promoter region of the LDHA, NLRP3, and IL-1β genes. Furthermore, our in vivo experiments showed that Sal B has a good effect on alleviating CCl_4_-induced liver injury. An examination of liver tissues and the Kupffer cells isolated from those tissues proved that Sal B affects the M1 polarization of macrophages and the level of histone lactylation. Together, our data reveal that Sal B has a potential mechanism of inhibiting the histone lactylation of macrophages by downregulating the level of LDHA in the treatment of liver injury.

## 1. Introduction

*Salvia miltiorrhiza* Bunge (SM) is one of the most widely used medicinal plants in traditional Chinese medicine, with its roots being an important part. SM was initially documented in the Shen Nongs Classic of Materia Medica and has been historically employed for the purpose of enhancing blood circulation and alleviating pain. The active ingredients in SM are mainly divided into liposoluble compounds and water-soluble compounds. Phenolic acid is the main bioactive, water-soluble compound in SM. Among the water-soluble active monomers present in SM, salvianolic acid B (Sal B) stands out as the most prevalent, exhibiting numerous pharmacological properties, including anti-inflammatory [1] and anti-organ injury and fibrosis effects [2,3].

The process of macrophage activation from an inactivated (M0) status to an anti-infection macrophage (M1) status is accompanied by the reprogramming of glycolytic metabolism. The most typical characteristic of this metabolic reprogramming of macrophages is the switching from a low glycolytic efficiency to a high glycolytic efficiency [4]. Glycolysis, the metabolic pathway responsible for converting glucose into pyruvate, is predominantly utilized by cells to generate lactate under normal oxygenation conditions, a phenomenon commonly referred to as “aerobic glycolysis” [5]. Numerous studies have provided evidence showcasing the significance of various pivotal enzymes in glycolysis, including hexokinase 2 (HK2) [6], M2-type pyruvate kinase (PKM2) [7], and lactate dehydrogenase A (LDHA) [8], which can influence the inflammatory response of macrophages. The existing mechanism studies focus on how these enzymes affect glucose uptake and downstream metabolism, which, in turn, ultimately affect the secretion of cellular inflammatory mediators. During glycolysis, the key rate-limiting enzyme LDHA converts pyruvate to lactate [9], and the lactate produced through this pathway is called “endogenous lactate”. It has been shown that macrophages subjected to lipopolysaccharide (LPS) stimulation can cause an increase in glycolysis, which ultimately leads to an elevated concentration of “endogenous lactate” [10].

Recent breakthrough research found that lactate acts as a precursor to the apparent lactylation of histone lysine residues, which can regulate gene transcription in chromatin [11]. Lactate is an important product in glycolysis and was once considered a metabolic waste product, but in recent years it has been found to play an important regulatory role in immune cells [12]. The modification of histone lactylation has been shown to be a novel epigenetic event in macrophages [11]. The content of lactate is increased in M1 macrophages after the invasion of bacterial endotoxins such as LPS, and the level of histone lactylation modification is then elevated [13]. Therefore, exploring the role of histone lactylation in macrophage inflammation is particularly important for the identification of new potential therapeutic targets.

Sal B can regulate ischemia and reperfusion cardiac macrophage polarization by interfering with the macrophage glycolytic process [14]. Sal B demonstrates significant efficacy in suppressing respiratory inflammation in mice afflicted with asthma [15]. Macrophages play an important role in liver injury. It has been reported that Sal B can alleviate liver injury and fibrosis through oxidative stress-related pathways [16,17,18,19]. However, the relevant effects of Sal B on histone lactylation are still not elucidated.

The purpose of this study was to analyze the mechanisms whereby Sal B downregulates the production of lactate by blocking LDHA, followed by further inhibition of histone lactylation, thereby inhibiting the associated inflammatory pathway to suppress macrophage inflammation, as well as polarization. Moreover, we also examined the pharmacological effect of Sal B on CCl_4_-induced liver injury due to the fact that M1 macrophages play a significant role in hepatitis, hepatic fibrosis, and hepatic cirrhosis. According to the results, Sal B targets the LDHA protein to interfere with the production of lactate, following the induction of a low level of histone lactylation in M1 macrophages, and the results were also confirmed in a mouse model of CCl_4_-induced liver injury.

## 2. Results

### 2.1. Sal B Reduces the Inflammatory Responses and the Polarization of Macrophages Stimulated by LPS

**Figure 1:**
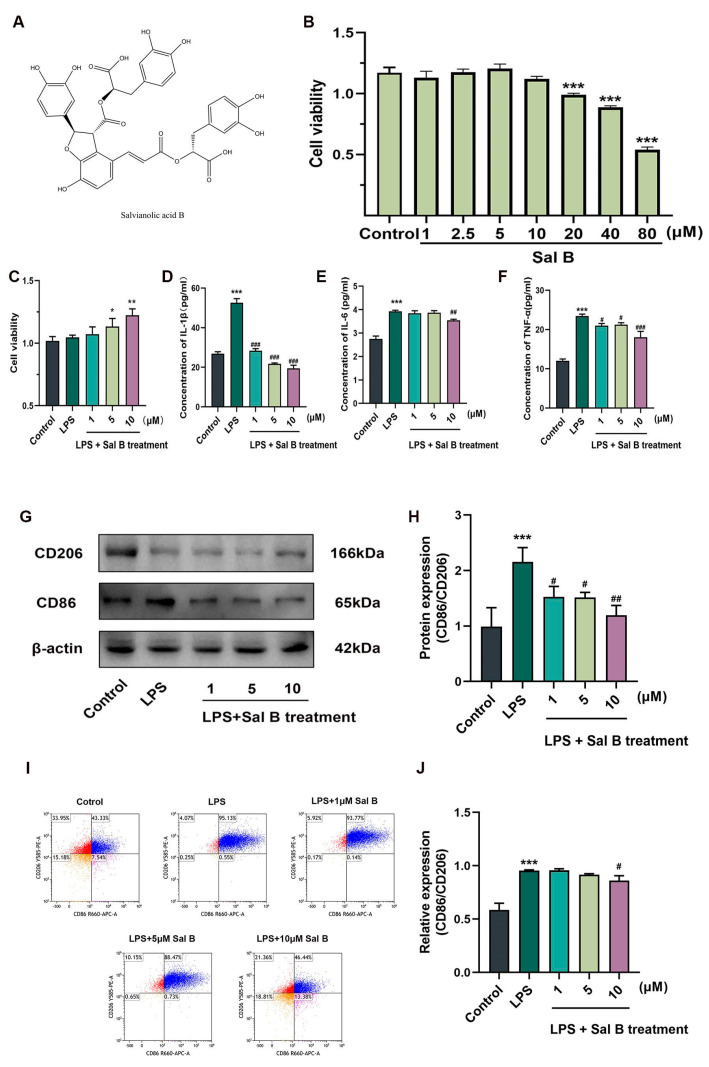
Sal B reduces M1 macrophage inflammatory responses and polarization. (**A**) The chemical structure of Sal B. (**B**,**C**) CCK8 was used to determine the viability of RAW264.7 cells treated with Sal B and LPS at indicated concentrations. Concentrations of (**D**) IL-1β, (**E**) IL-6, and (**F**) TNF-α levels were treated with LPS (1 μg/mL), 2-DG (10 mM) and Sal B (1, 5, 10 μM). (**G**) The protein expression of CD86 and CD206 in cells treated with LPS, 2-DG, and Sal B for 24 h. β-actin was used as the loading control. (**H**) Quantitative image analysis of (**G**). (**I**) Flow cytometry analysis of CD86 and CD206. (**J**) Quantitative image analysis of (**I**). All data are shown as the mean ± SD. * *p* < 0.05, ** *p* < 0.01, and *** *p* < 0.001 vs. the control group. # *p* < 0.05, ## *p* < 0.01, and ### *p* < 0.001 vs. the LPS group, *n* = 6 for test kit detection and *n* = 3 for protein detection.

In this study, RAW264.7 cells were stimulated with lipopolysaccharide (LPS) to create an in vitro model of M1 macrophages. The objective was to evaluate the potential therapeutic effects of Sal B on inflammatory responses. The structural formula of salvianolic acid B is shown in Figure 1A. The dosages of Sal B were 1, 5, and 10 μM according to the CCK8 experiment (Figure 1B,C). After LPS stimulation, the expression of inflammatory factors and polarization indicators of macrophages increased. And then we examined the inhibitory effect of Sal B on inflammatory cytokines. Subsequently, we investigated the inhibitory impact of Sal B on inflammatory cytokines. Sal B treatment resulted in a significant dose-dependent decrease in IL-1β levels (Figure 1D), whereas the expression of IL-6 and TNF-α only exhibited a decrease in the high-dose group (Figure 1E,F). We also examined the indicators related to macrophage polarization. Figure 1G,H shows that the expression of CD86 was decreased with the elevation of the Sal B dose, whereas CD206 did the opposite. Furthermore, we incorporated flow cytometry as an additional method to assess the membrane expression of CD86 and CD206, and the findings exhibited a general agreement with the outcomes obtained from western blot analysis (Figure 1I,J). Taken together, Sal B could reduce macrophage inflammation and M1 polarization.

### 2.2. Sal B Inhibits LPS Induced Glycolysis and NLRP3 in Macrophages

**Figure 2:**
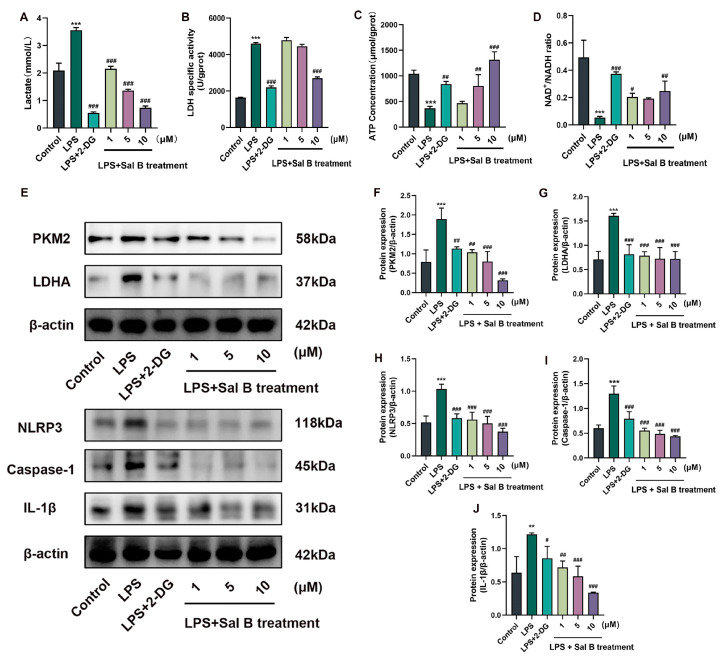
Sal B inhibits glycolysis and NLRP3 inflammasome in macrophages. (**A**) lactate concentration of cell supernatant, (**B**) LDH specific activity levels; (**C**) ATP concentration of cell lysate; and (**D**) NAD+/NADH ratio of cell lysate were treated with LPS, 2-DG and Sal B for 24 h. (**E**) The cellular protein expression levels of PKM2, LDHA, NLRP3, caspase-1, and IL-1β were assessed after a 24 h treatment with LPS, 2-DG, and Sal B. β-actin was used as the loading control. (**F**–**J**) Quantitative image analysis of (**E**). All data are shown as the mean ± SD. ** *p* < 0.01, and *** *p* < 0.001 vs. the control group. # *p* < 0.05, ## *p* < 0.01, and ### *p* < 0.001 vs. the LPS group, *n* = 6 for test kit detection and *n* = 3 for protein detection.

After LPS stimulation, the glycolysis level of macrophages increased. During LPS stimulation, Sal B and the 2-DG group could downregulate the indicators of glycolysis, as well as lowering the content of lactate (Figure 2A) and inhibiting the activity of LDH (Figure 2B), combined with increasing the content of ATP (Figure 2C) and the ratio of NAD+/NADH (Figure 2D). To further confirm these results, we conducted an investigation into the expression of LDHA, a key glycolytic protein, and its upstream protein, PKM2. Additionally, we examined the expressions of NLRP3, caspase-1, and IL-1β inflammatory proteins, as depicted in Figure 2E. The statistical analysis is presented in Figure 2F–J. The presence of Sal B resulted in a decrease in the expression of PKM2, LDHA, NLRP3, caspase-1, and IL-1β. These findings suggest that Sal B inhibits LPS-induced glycolysis and the activation of NLRP3 in macrophages.

### 2.3. LDHA Overexpression Impairs the Inhibiting Effects of Sal B on Glycolysis, NLRP3, and M1 Polarization in LPS-Stimlated Macrophages

**Figure 3:**
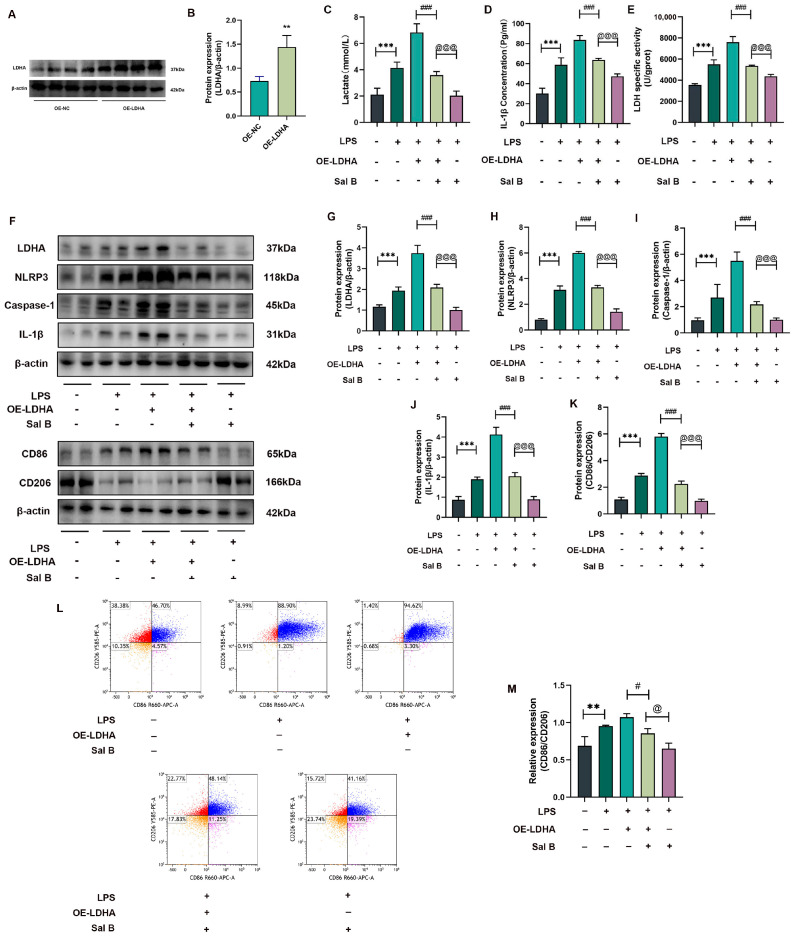
Sal B inhibits glycolysis, NLRP3 inflammasome, and polarization in macrophages via LDHA overexpression. (**A**) Western blot analysis of LDHA levels in negative control (NC) and LDHA-overexpressed RAW264.7 cells. (**B**) Quantitative image analysis of (**A**). (**C**) Lactate concentration of cell supernatant, (**D**) LDH specific activity, and (**E**) various concentrations of IL-1β were treated with LDHA overexpression for 8 h, and then they were treated with LPS and Sal B for 24 h. (**F**) The protein expression levels of LDHA, NLRP3, caspase-1, IL-1β, CD86, and CD206 were examined after treatment with LDHA overexpression for a duration of 8 h, followed by treatment with LPS and Sal B for a period of 24 h. β-actin was used as the loading control. (**G**–**K**) Quantitative image analysis of (**F**). (**L**) Flow cytometry analysis of CD86 and CD206. (**M**) Quantitative image analysis of (**J**). All data are shown as the mean ± SD. ** *p* < 0.01 and *** *p* < 0.001 vs. the NC group. # *p* < 0.001 and ### *p* < 0.001 vs. the LPS + OE-LDHA group. @ *p* < 0.001 and @@@ *p* < 0.001 vs. the LPS+Sal B group; *n* = 6 for test kit detection, and *n* = 4 for protein detection.

We first verified the successful construction of the LDHA overexpression plasmid and performed statistical analysis (Figure 3A,B). Sal B could reduce the concentration of lactate, the activity of LDH, and the content of IL-1β in LDHA-overexpressed macrophages. However, the inhibiting effects significantly weaken compared with NC macrophages (Figure 3C–E). Consistently, the debilitating effects of Sal B downregulation were observed in LDHA, NLRP3, caspase-1, IL-1β protein expression, and the polarization index CD86/CD206 protein expression (Figure 3F). CD86/CD206 ratios were detected by flow cytometry, which is consistent with the above results (Figure 3L,M). All of these protein bands were quantified and statistically analyzed (Figure 3G–K). Altogether, we confirmed the mechanisms of Sal B reduce glycolysis, NLRP3, and M1 polarization are partly associated with the inhibitory effect on LDHA.

### 2.4. Sal B Regulated Histone Lactylation and Binding Ability of H3K18la to LDHA, NLRP3 and IL-1β Genes

**Figure 4:**
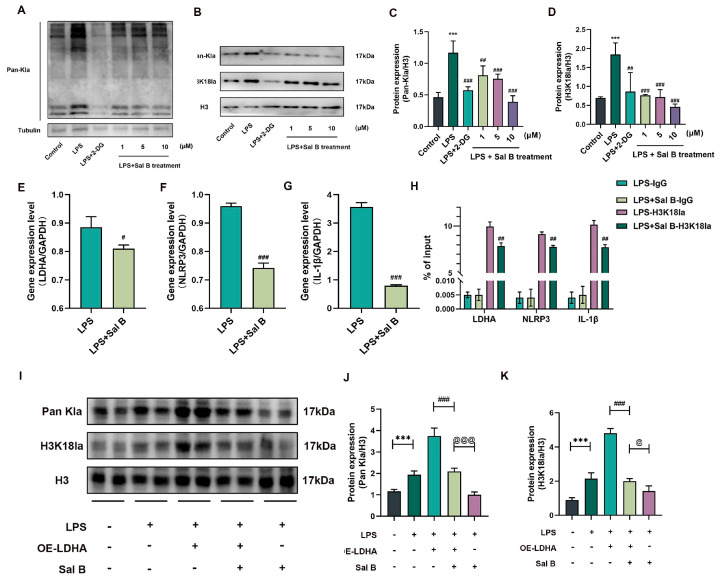
Sal B regulated binding ability of H3K18la to related genes. (**A**) The protein expression of total Pan Kla was treated with LPS, 2-DG, and Sal B for 24 h. (**B**) The protein expression of Pan Kla and H3K18la was treated with LPS, 2-DG, and Sal B for 24 h. (**C**,**D**) Quantitative image analysis of (**B**). (**E**–**G**) The gene expression of LDHA, NLRP3, and IL-1β were treated with LPS and Sal B for 24 h. (**H**) ChIP-qPCR to confirm changes in H3K18la modification of the indicated genes. (**I**) The protein expression of Pan Kla and H3K18la was treated with LDHA overexpression for 8 h, and then treated with LPS and Sal B for 24 h. (**J**,**K**) Quantitative image analysis of (**I**). *** *p* < 0.001 vs. the control or NC group. # *p* < 0.05, ## *p* < 0.01, and ### *p* < 0.001 (**C**–**H**) vs. the LPS group and (**J**,**K**) vs. the LPS+OE-LDHA group. @ *p* < 0.001. @@@ *p* < 0.001 vs. the LPS + Sal B group. *n* = 6 for test kit detection and *n* = 3–4 for protein detection.

Initially, we confirmed that the level of lactatation modification in macrophages increases after LPS stimulation, and we conducted an investigation to assess the impact of Sal B and 2-DG on the overall protein lactylation of macrophages, employing the method of total protein extraction (Figure 4A). To further explore the downstream mechanism caused by lactate changes, we examined changes in the histone Pan Kla and the lactate of H3K18la in RAW264.7 cells treated with Sal B. The findings of the study indicate that Sal B and 2-DG has the activity to decrease the expression of Pan-Kla and H3K18la (Figure 4B). The statistical analysis is shown in Figure 4C,D. Furthermore, we performed Chip-qPCR and qRT-PCR experiments to verify the binding ability of H3K18la to the differential genes. Additionally, it was observed that a concentration of 10 μM Sal B can hinder the binding capacity of H3K18la to LDHA, NLRP3, and IL-1β genes (Figure 4E–H). Furthermore, the researchers conducted an experiment where LDHA was overexpressed in LPS-stimulated macrophages, and it was discovered that the inhibitory effects of Sal B on the expression of Pan-Kla and H3K18la were compromised (Figure 4I). The statistical analysis is shown in Figure 4J,K.

### 2.5. Sal B Exerts Anti-Fibrosis Effects on CCl_4_-Induced Liver Injury

**Figure 5:**
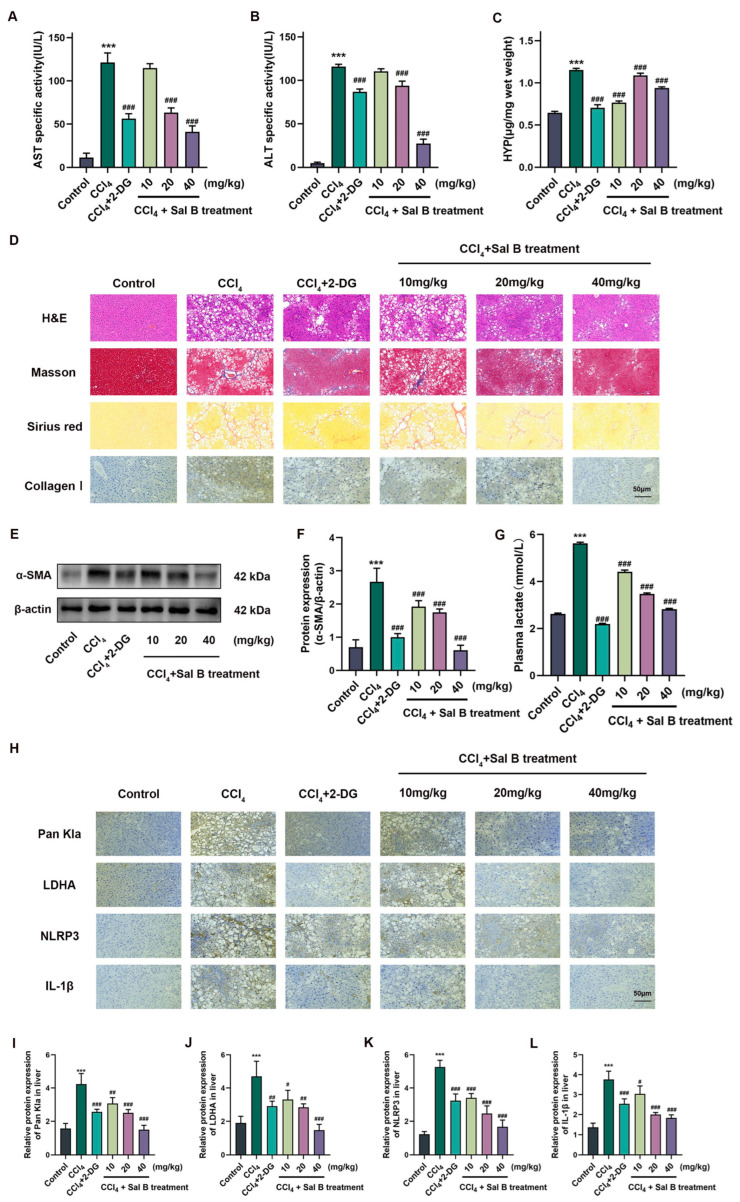
Sal B exerts anti-fibrosis effects on liver injury. Concentrations of (**A**) AST, (**B**) ALT, and (**C**) HYP were treated with CCl_4_ (0.1 mL/10 g), 2-DG (100 mg/kg), and Sal B (10, 20, 40 mg/kg). (**D**) Hematoxylin and eosin (H&E), Masson, Sirius red, and collagen Ⅰ of histological examination (scale bar = 50 mm). (**E**) The protein expression of α-SMA was treated 2-DG and Sal B in mice. β-actin was used as the loading control. (**F**) Quantitative image analysis of (**E**). (**G**) Concentrations of plasma lactate were treated 2-DG and Sal B in mice. (**H**) Immunohistochemistry staining of Pan Kla, LDHA, NLRP3, and IL-1β were treated 2-DG and Sal B in mice (scale bar = 50 mm). (**I**–**L**) Quantitative image analysis of (**H**). All data are shown as the mean ± SD. *** *p* < 0.001 vs. the control group. # *p* < 0.05, ## *p* < 0.01, and ### *p* < 0.001 vs. the CCl_4_ group, *n* = 6 for test kit detection and *n* = 3 for protein detection.

In order to investigate the potential regulatory effect of Sal B in vivo, a mouse model of CCl_4_-induced liver injury was utilized to examine the role of M1 macrophages in liver injury. Initially, mouse models of CCl_4_-induced liver injury were evaluated to confirm the model establishment, and the pharmacodynamic effects of Sal B on this model were determined. The results of our study demonstrated that Sal B, particularly in the medium and high dose groups, exhibited a noteworthy inhibitory effect on liver injury in CCl_4_-induced mice. Additionally, Sal B and 2-DG administration led to a reduction in cholestasis, infiltration of inflammatory cells, hemorrhagic necrosis, as well as decreased levels of ALT and AST. (Figure 5A,B,D). In order to conduct a more comprehensive examination of liver disease, the fibrosis-related indices were evaluated. The administration of Sal B and 2-DG in the specified dosage group significantly reduced the deposition of extracellular matrix, and the hydroxyproline (HYP) test provided additional evidence that the excessive production of collagen Ⅰ was eliminated following treatment with Sal B and the 2-DG control group (Figure 5C,D). Additionally, Sal B and 2-DG suppressed the expression of collagen Ⅰ and α-SMA. (Figure 5D–F). These findings indicate that Sal B effectively safeguarded against liver injury and fibrosis induced by CCl_4_.

### 2.6. Sal B Reduces Lactylation in Liver Tissues in Mice Stimulating CCl_4_

In the CCl_4_-induced liver injury mice model, the effect of Sal B and 2-DG on plasma lactate was examined (Figure 5G), while the expression of Pan Kla, LDHA, NLRP3, and IL-1β proteins was also detected and quantitatively analyzed by immunohistochemistry (Figure 5H–L).

### 2.7. Sal B Reduces Mechanisms Related to Histone Lactylation in Kupffer Cells

**Figure 6:**
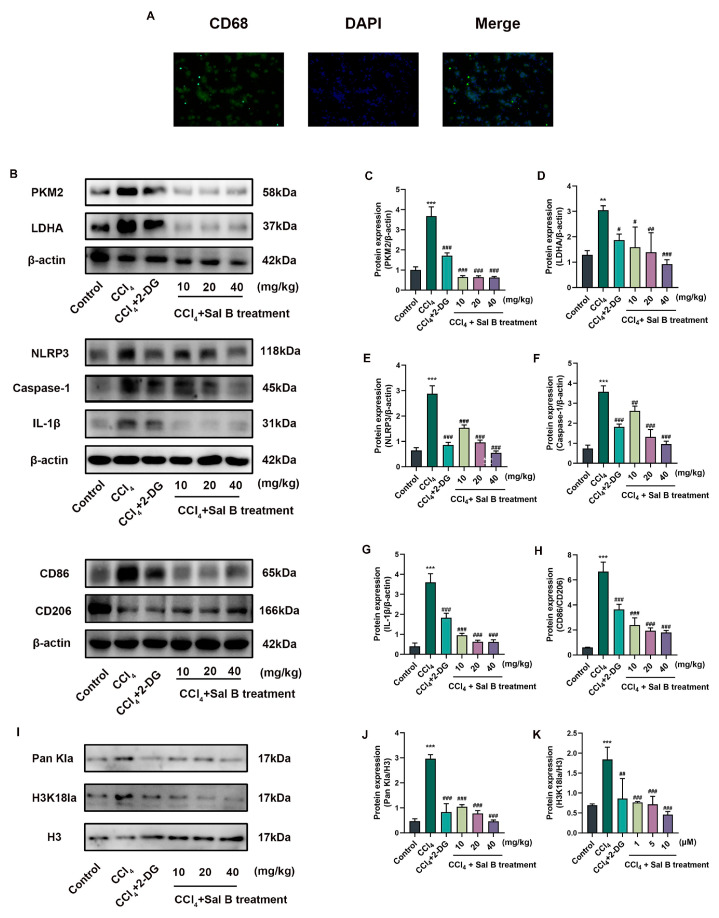
Sal B reduces histone lactylation in Kupffer cells. (**A**) CD68 immunofluorescence staining was used to identify Kupffer cells extracted from liver (scale bar = 50 mm). (**B**) The protein expression of PKM2, LDHA, NLRP3, caspase-1, IL-1β, CD86, and CD206 was treated 2-DG and Sal B in Kupffer cells. β-actin was used as the loading control. (**C**–**H**) Quantitative image analysis of (**B**). (**I**) The nucleoprotein expression of Pan Kla and H3K18la was treated 2-DG and Sal B in Kupffer cells. Histone H3 was used as the loading control. (**J**,**K**) Quantitative image analysis of (**I**). All data are shown as the mean ± SD. ** *p* < 0.01, and *** *p* < 0.001 vs. the control group. # *p* < 0.05, ## *p* < 0.01, and ### *p* < 0.001 vs. the CCl_4_ group, *n* = 3 for protein detection.

Kupffer cells were isolated at the end of the Sal B and 2-DG interventions, and the result of CD68 immunofluorescence staining proved that most of the extracted cells were Kupffer cells (Figure 6A). Kupffer cells exhibited comparable outcomes to LPS-induced RAW264.7 cells under CCl_4_ stimulation. Sal B and the 2-DG control group decreased the elevation of histone lactate modification caused by CCl_4_-induced liver injury, and they also decreased the expression of the glycolytic and inflammatory cytokine-related markers PKM2, LDHA, NLRP3, caspase-1, and IL-1β in these isolated Kupffer cells (Figure 6B–H). Meanwhile, Sal B and 2-DG could also reduce histone lactylation modification and H3K18la in detected Kupffer cells (Figure 6I–K). These results suggest that Kupffer cells also undergo glycolysis, NLRP3 inflammasome, and histone lactylation in liver injury, and Sal B’s effect on liver injury may be achieved by reducing histone lactylation in Kupffer cells.

## 3. Discussion

It is well known that macrophages are polarized into different phenotypes, the M1 or M2 type, in different immune microenvironments. Stimulation with LPS is the classical induction method for M1 macrophages. The characteristic markers of M1 macrophages include elevated levels of pro-inflammatory cytokines, such as IL-1β, TNF-α, and IL-6, as well as the presence of the membrane protein CD86 [20,21]. We opted to measure the CD86-to-CD206 total protein ratio in RAW264.7 cells, as it provides insight into the polarization level of macrophages. M1 macrophages promote inflammatory responses, which are associated with many types of tissue injury. Damage-associated molecular patterns (DAMPs), such as lipopolysaccharide (LPS), have the ability to activate hepatic macrophages and induce the release of inflammatory cytokines. These cytokines subsequently contribute to the maintenance of the inflammatory state. In this study, we examined the regulatory impact of Sal B on M1 macrophages and confirmed its efficacy in vivo using a CCl_4_-induced liver injury model.

Previous literature has reported that Sal B inhibits M1 macrophage polarization by suppressing NF-κB pathway activation and reducing Akt/mTOR activation [22]. In this paper, we revealed the potential mechanism by which Sal B inhibits macrophages trend toward M1 type from the perspective of the regulation of glycolysis and histone lactylation.

Glycolysis, which is characterized by excessive lactate production under aerobic deterioration, was previously thought to be a unique metabolic feature of tumor cells, but researchers found that this effect also exists in the processes of macrophages and other immune cells [23]. Under normal conditions, the glucose metabolism of M0 macrophages is in a state of balance between glycolysis and the tricarboxylic acid cycle (TCA). However, it has been observed that the energy metabolism in M1-polarized macrophages undergoes a shift towards glycolysis in comparison to M0 macrophages [24]. 2-DG is a recognized glycolysis inhibitor that inhibits glycolysis [25]. Consequently, 2-DG has been chosen as a positive control drug in research pertaining to glycolysis.

The existing literature has shown that PKM2 and LDHA can regulate glycolysis, affecting the polarization function of macrophages and interfering with liver fibrosis [26]. The regulation of glucose metabolism is facilitated by Sal B through the inhibition of the PI3K/AKT/HIF-1α inflammatory signaling pathway [27]. In both RAW264.7 cells and Kupffer cells, Sal B is proven to downregulate the level of glycolysis for the data we detect in this paper. The release of LDH and the secretion of IL-1β serve as significant markers for the occurrence of pyroptosis in macrophage inflammation [28]. LDHA, an isoform of LDH, is a key rate-limiting enzyme in the final stage of cellular glycolysis, catalyzing the redox reaction of NADH and pyruvate to produce lactate and NAD+, in which the LDHA subunit plays a key role in glycolysis [29]. It is worth noting that Sal B exhibits inhibitory effects on the expression of the LDHA protein. Meanwhile, the process of glycolysis is also accompanied by changes in ATP energy. When LPS stimulates macrophages to increase the expression of glycolysis, ATP production decreases [30]. The NAD+/NADH ratio and ATP content have been employed as fundamental indicators for assessing glycolysis [31]. The role of LDHA in immune cells involves facilitating the generation of lactate as a means of supplying energy to the cells, thereby establishing itself as a crucial focal point for glycolysis [32]. Here, we investigated the trends of the effects of Sal B’s inhibition of inflammation and glycolysis in RAW264.7 cells while LDHA is overexpressed. The observed mitigation of Sal B’s effects in response to LDHA overexpression provides compelling evidence that inhibiting LDHA is a key mechanism underlying the action of Sal B.

Historically regarded as a metabolic waste product, lactate has recently been discovered to exert significant regulatory influence on immune cells [33]. In 2019, Zhang et al. reported that lactate could be used as a precursor to the apparent lactation of histone lysine residues, which is associated with the transcription of genes in chromatin [16]. Histone, a protein on chromatin, is entangled by DNA to form nucleosomes, which play an important role in gene transcription. The modification of histones by lactate through lactyl coenzyme A drives histone lactylation [34]. This lactate-induced histone lactylation has been reported to occur in M1 macrophages, specifically at the histone H3K18 modification site [14]. It has been reported that the intervention of lactate could promote the production of the NLRP3 inflammasome [35]. The NLRP3 inflammasome complex, which has been widely studied, is composed of a series of protein complexes, including NLRP3 and caspase-1 [36]. When macrophages are subjected to LPS stimulation, the activated NLRP3/caspase-1 complex also activates the downstream pro-inflammatory factor IL-1β [37]. Similarly, LDHA has also been revealed to stimulate the NLRP3 inflammasome through the accumulation of lactate [13]. In this study, we associated this process with histone lactylation modification through ChIP-qPCR and qRT-PCR experiments. The overexpression of LDHA directly increases endogenous lactate production, which, in turn, affects histone lactylation modifications and NLRP3 inflammatory vesicle-associated pathway protein expression.

Next, we found that Sal B regulated histone lactylation modifications and H3K18la expression in both RAW264.7 and Kuppfer cells. Furthermore, a total of 289 lactylation sites were detected among 181 proteins that underwent lactylation modifications in Kupffer cells [38]. Pyroptosis is a regulated inflammatory modality of cellular demise distinguished by cellular enlargement, lysis, and the liberation of intracellular constituents [39]. Moreover, we revealed that Sal B reduced the expression of H3K18la sites and the downstream NLRP3/caspase-1/IL-1β inflammatory pathway in M1 macrophages. Sal B plays an important role in anti-liver injury, such as hepatitis and fibrosis [40]. The pathological process of liver fibrosis is long-term and is often accompanied by an inflammatory response caused by macrophage polarization [41]. In addition, the pathogenesis of liver disease is closely related to Kupffer cells, which are macrophages that settle in the liver. Kupffer cells are specialized macrophages of the liver that are present in the blood sinuses and act as the first line of defense in the liver against foreign molecules, along with intestinal antigens excreted through the portal vein [42], and they are critical for the liver and the system’s response to the pathogen [43]. The M1 polarization of Kupffer cells could purpose the process of liver injury and fibrosis [44].

To explore the role of drug Sal B in macrophage polarization for regulating histone lactylation modifications, we performed RNA-seq, ChIP-qPCR and qRT-PCR experiments. In the RNA-seq experiments, we found that gene expression was closely related to glycolysis after the Sal B intervention in M1 macrophages. qRT-PCR was conducted to confirm this result. Histone lactylation modifications usually regulate the process of glycolysis or inflammation through gene transcription [45]. ChIP-seq and CUT&Tag assays were employed to ascertain the binding affinity of lactate modification towards relevant genes [46,47]. Notably, the presence of H3K18 lactylation in pulmonary fibrosis was found to enhance the transcription of YTHDF1 [48]. We found via ChIP-qPCR experiments that Sal B can reduce the levels of H3K18la in the promoter region of the M1 macrophage glycolysis-related gene LDHA and the inflammation-related genes NLRP3 and IL-1β, suggesting that Sal B can reduce endogenous lactate-mediated histone lactylation modifications in M1 macrophages, which, in turn, upregulates the expression of genes.

In the past few years, histone lactylation has been introduced as a newly identified modification and has been strongly linked to various fibrotic conditions [48,49,50,51]. Similar to liver fibrosis, histone lactylation modifications are elevated in both alveolar macrophages of pulmonary fibrosis and placental fibrosis [49,52]. The activation of hepatic stellate cells, a key process in liver fibrosis, also involves histone lactylation modification, specifically with increased levels of H3K18la [52]. It has been reported in the liver that mitochondrial pyruvate carrier 1 (MPC1) regulates fatty acid synthase lactylation, affecting both liver fibrosis and nonalcoholic fatty liver [51]. In this study, we verified the key histone lactylation mechanism discussed in the LPS-induced RAW264.7 macrophage model in the CCl_4_-induced mouse liver injury model, and found that similar histone lactylation occurred in Kupffer cells in the liver injury model mice. These results revealed that the level of histone lactylation in Kupffer cells increase during the liver injury process and that Sal B could reduce protect against this course, thus inhibiting the M1 polarization of Kupffer cells and ultimately reducing the liver injury induced by CCl_4_ in mice.

## 4. Materials and Methods

### 4.1. Chemical and Reagents

2-deoxy-D-glucose (2-DG) (HY-13966) was obtained from MedChemExpress. Collagenase type IV (V900893) and LPS (L8274) were obtained from Sigma-Aldrich (Shanghai, China). Sal B (PS0054-0020, 98.0%) was obtained from Chengdu Push Bio-Technology (Chengdu, China). Lactic acid assay kit (A019-2-1) and ATP assay kit (A095-1-1) were obtained from Nanjing Jiancheng Bioengineering Institute (Nanjing, China). NAD+/NADH (S0175) assay kit was obtained from Beyotime Biotechnology (Beijing, China). Enhanced cell counting kit 8 (WST-8/CCK8, E-CK-A362), AST/GOT (E-BC-K236-M), and ALT/GPT (E-BC-K235-M) activity assay kits, ELISA kit, hydroxyproline (HYP, E-BC-K062-S) colorimetric assay kit, and lactate dehydrogenase (LDH, E-BC-K046-M) activity assay kit were obtained from Elabscience (Wuhan, China). Interleukin 1β (IL-1β, EK201BHS), tumor necrosis factor α (TNF-α, EK282HS), and interleukin 6 (IL-6, EK206HS) were obtained from MULTISCIENCES (Hangzhou, China).

### 4.2. Cell Culture

RAW264.7 cells (CL-0190) and special medium (CM-0190) were purchased from Pricella (Wuhan, China). The cells were blown down with a pasteurized straw, inoculated into a 6-well plate at a density of 5 × 105 cells per well, and incubated at 37 °C with 5% CO_2_. The cells were cultured at 37 °C for 24 h, induced with 1 μg/mL LPS for 24 h, treated with Sal B (1, 5, and 10 μM) for 24 h, and then harvested.

### 4.3. Animals

Sixty male ICR mice (license No. LL2023022214) (SPF, 18–22 g) were obtained from Slack Jingda Experimental Animal Company (Changsha, China) and given free access to food and water under a light-dark cycle. According to the Guide for the Care and Use of Laboratory Animals published by the National Institutes of Health, the experiment was approved by the Institutional Ethical Committee on Animal Care and Experimentations of Hunan University of Chinese Medicine. After one week of adaptive feeding, all mice were randomly divided into six groups (*n* = 10 mice per group): (1) The control group; (2) the model group; (3) the positive group (2-DG, 100 mg/kg/d); (4) low-dose Sal B group (10 mg/kg/d); (5) middle-dose Sal B group (20 mg/kg/d); and (6) high-dose Sal B group (40 mg/kg/d). In groups 2 to 6, 20% CC1_4_ (dissolved in corn oil) was injected subcutaneously at a dose of 0.1 mL/10 g three times per week for six weeks [53]. The mice belonging to group 1 were administered isopycnic corn oil in a uniform manner through injection. Mice in the control group and the model group were given normal saline by intragastric administration. Mice in the positive control group were subjected to 100 mg/kg of glycolysis inhibitor 2-DG by intragastric administration according to 0.2 mL/20 g. Sal B was administered in doses of 10, 20, and 40 mg/kg in low, medium, and high dose groups, respectively. All animals were given intragastric doses of volume 0.2 mL/20 g once a day for 6 weeks.

### 4.4. Western Blot Analysis

The cells or liver tissues were lysed in RIPA lysis buffer (WB3100, New Cell & Molecular Biotech Co., Suzhou, China) containing protease and phosphatase inhibitors for 30 min, and then centrifuged at 13,000 rpm at 4 °C for 10 min. The supernatant was obtained, and the protein concentration was determined by a BCA kit (WB6501, New Cell & Molecular Biotech Co., Suzhou, China) Briefly, 20–80 μg protein extracts were separated by 10–15% SDS-polyacrylamide gel. PVDF membrane was used for transmembrane, then sealed with skim milk or rapid sealing solution, and overnight with primary antibody at 4 °C. CD86 (1:1000, 26903-1-AP), CD206 (1:1000, 18704-1-AP), PKM2 (1:1000, 15822-1-AP), LDHA (1:5000, 19987-1-AP), NLRP3 (1:5000, 68102-1-Ig), α-SMA (1:20000, 67735-1-Ig), and β-actin (1:10000, 81115-1-RR) were obtained from Proteintech (Wuhan, China). Caspase-1 (1:1000, ET1608-69) was obtained from HUABIO (Hangzhou, China). IL-1β (1:1000, A16288) was obtained from ABclonal (Wuhan, China). Pan-Kla (1:1000, PTM-1401), H3K18la (1:1000, PTM-1406RM), and histone H3 (1:1000, PTM-6613) were obtained from PTM BIO (Hangzhou, China). The secondary antibody was incubated at room temperature for 1 h and analyzed with ECL chemiluminescence substrate. The majority of membranes were stripped with the stripping liquid (WB6500, New Cell & Molecular Biotech Co., Suzhou, China) for 30 min, and then the above steps were repeated to incubate the antibody and analyze it with an ECL chemiluminescence substrate. A few membranes were cut horizontally. All control bands at different time points are not included in the representative Western blot images. The data were analyzed using ImageJ 1.8.0.

### 4.5. Flow Cytometry

Flow cytometry was employed to assess the expression of surface markers on stimulated cells. A total of 1 × 107 cells/mL of RAW264.7 cells were collected and subjected to ice-cold PBS buffer washing prior to the addition of allophycocyanin (APC)-conjugated anti-CD86 antibody. The samples were then incubated in darkness at 25 °C for 15 min. To disrupt the cell membranes, a permeabilization wash buffer was introduced at room temperature. The sample containing phycoerythrin (PE)-conjugated anti-CD206 antibody was incubated in darkness at 25 °C for an additional 15 min, followed by subsequent washing and resuspension in PBS containing 0.1% BSA. After pipetting and mixing, the surface markers were detected via a Cytomics FC 500 flow cytometer (Beckman Coulter, Brea, CA, USA).

### 4.6. Histological Analysis

The histological examination of liver tissue samples involved staining with hematoxylin and eosin (H&E), Masson, and Sirius red. Each microscopic field was examined under a light microscope.

### 4.7. Kupffer Cell Extraction

The liver tissues were cut with scissors and placed in a petri dish, washed three times with PBS, followed by digestion with 1 mg/mL type IV collagen (Sigma-Aldrich, V900893), and kept at 4 °C overnight. The cells were transferred to a centrifuge tube with a 100 μm cell filter and centrifuged twice at 400 g/5 min. Forty-five percent Percoll (Solarbio, P8370) was added along the tube wall successively and centrifuged at 900 g/15 min. The white precipitates at the bottom were Kupffer cells. Following the extraction of Kupffer cells, the cells were fixed with 4% paraformaldehyde and blocked with 5% BSA. Subsequently, the cells were incubated overnight at 4 °C with the primary antibody CD68 (DF7518, Affinity). Afterward, the sample was incubated with a fluorescent secondary antibody for 1 h. Following a washing step, the nucleus of the sample was re-stained with DAPI. The results were observed using fluorescence microscopy. Kupffer cells from two mice were pooled for each sample. The assay was repeated in triplicate for each sample.

### 4.8. Immunohistochemistry Analysis

The liver tissues were rinsed with sterile saline solution and subsequently treated with 4% paraformaldehyde for a duration of 24 h. Subsequent steps involved the utilization of a highly sensitive and expeditious immunohistochemical kit (E-IR-R221, Elabscience, Wuhan, China). For semi-quantitative analysis of liver tissues, the Image J 1.8.0 scoring system was employed. Three distinct visual fields were randomly chosen from each group to ascertain the proportion of positively stained areas using the ImageJ 1.8.0 analysis software.

### 4.9. Cell Transfection

LDHA-overexpressed vectors (OE-LDHA) and their controls (OE-NC) were provided by Genechem (Shanghai, China). Cells were cultured for 8 h after transfection and then used for further experiments.

### 4.10. Chromatin Immunoprecipitation (ChIP) -qPCR

Thirty-seven percent formaldehyde solution was added into the cells so that the final concentration of formaldehyde was 1%, and the cells were incubated at room temperature for 10 min. The crosslinking reaction was terminated by adding 2.5 M glycine to each reaction system until the final concentration was 125 mM and incubated at room temperature for 5 min. One milliliter of the mixture of PBS + protease inhibitors was added and transferred to a 1.5 mL centrifuge tube. The sample was re-suspended with 300 μL of cracking buffer and placed on the ice for 30 min. The sample was shaken once every 5 min. The DNA in the sample is broken using ultrasound. After the completion of the ultrasound, the supernatant was obtained by centrifugation at 12,000× *g* and 4 °C for 5 min. The fragment size of the ultrasonic sample was determined by 2% agarose gel electrophoresis after 20 μL of samples was decrosslinked and purified. For the ChIP-qPCR assay, subsequent qRT-PCR was performed to quantify the ChIP-enriched DNA. The antibodies used for ChIP were anti-H3K18la (PTM-1427RM). ChIP-qPCR primer sequences are listed in Table 1.

### 4.11. Quantitative Real-Time PCR Analysis (qRT-PCR)

After treatment with LPS (1 μg/mL) and Sal B(10 μM) at 37 °C for 24 h, total RNA was extracted utilizing RNA extraction reagent kits in accordance with the manufacturer’s protocol. Reverse transcription was conducted employing a PrimeScript RT Reagent kit with 1 µg of total RNA. The reaction system, comprising the cDNA, forward and reverse primers, and the SYBR Green PCR master mix, had a volume of 20 µL. The analysis of all data was carried out utilizing the GAPDH gene expression as an internal reference. The specific primers can be found in Table 2. To ensure that any observed effects were not attributable to treatment, multiple reference genes were tested for each experiment.

### 4.12. Statistical Analysis

A one-way ANOVA followed by the Bonferroni test was used for multiple group comparisons with GraphPad Prism 8.0. The two-tailed Student’s *t*-test was used for comparisons between two groups. *p* < 0.05 was considered a significant difference.

## 5. Conclusions

**Figure 7:**
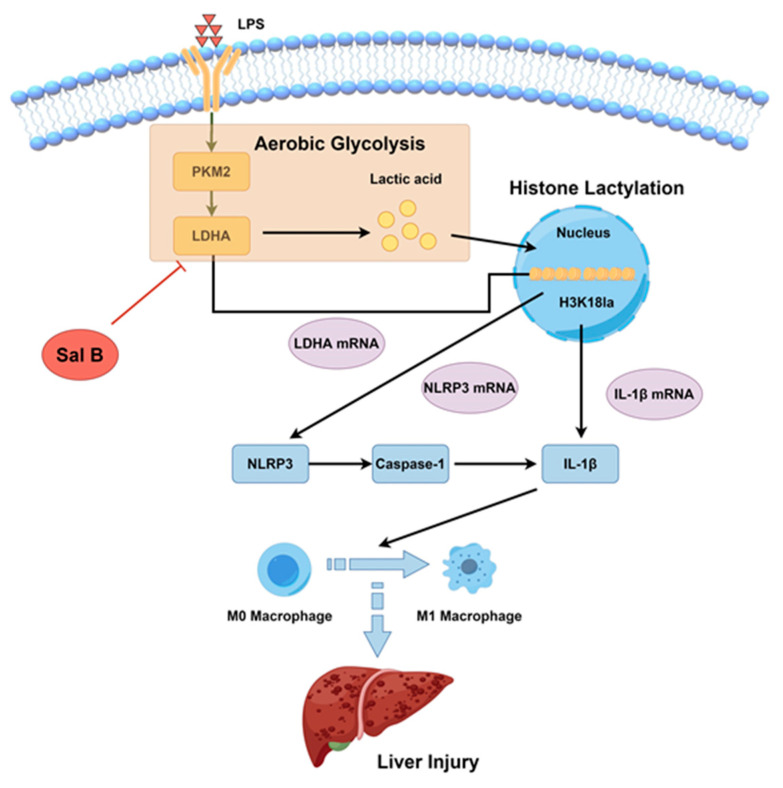
The potential mechanism whereby Sal B regulates lactate-mediated histone lactylation in macrophages in liver injury.

Our study suggests that Sal B inhibits the M1 polarization of macrophages by downregulating glycolytic metabolism. Downregulating the expression of LDHA is proved to be one of the mechanisms of the above effects. Both histone lactylation and the following NLRP3/caspase-1/IL-1β pathway are involved in this process. Moreover, Sal B is revealed to alleviate liver injury induced by CCl_4_. The detection of Kuppfer cells, isolated from Sal B-treated liver tissues, supports the effects of Sal B in vivo. Hence, it is anticipated that LDHA and H3K18la could serve as promising targets for the therapeutic intervention of macrophage polarization in the context of liver fibrosis, specifically in relation to the administration of Sal B (Figure 7).

## Figures and Tables

**Table 1:** Primer sequences for ChIP-qPCR.

Gene	Primer	Sequence (5′-3′)
Arg1-PTM:92bp	Forward	AAGCTGTGGCCTCAGAACAT
	Reverse	GGTAACCGCTGTGAAAGGAT
Arg1-HRE-2kb:85bp	Forward	TGTCTCTCCCAGTTTCCCCA
	Reverse	AGCAACTTGGCATCTGATGGA
gene desert:79bp	Forward	CTGCCAGGGTTGTAGAGAGG
	Reverse	GCCAGATCATATTGGCTTGG

**Table 2:** Primer sequences for real-time RT-PCR.

Gene	Primer	Sequence (5′-3′)
Mus GAPDH	Forward	ATGGGTGTGAACCACGAGA
	Reverse	CAGGGATGATGTTCTGGGCA
Mus LDHA	Forward	GTAACTGCGAACTCCAAGCT
	Reverse	CAAGCCACGTAGGTCAAGAT
Mus NLRP3	Forward	CCATCAATGCTGCTTCGACA
	Reverse	GAGCTCAGAACCAATGCGAG
Mus IL-1β	Forward	TCAGGCAGGCAGTATCACTC
	Reverse	AGCTCATATGGGTCCGACAG

## Data Availability

The data that support the findings of this study are available from the corresponding authors upon reasonable request.
